# Heterogeneity of D-Serine Distribution in the Human Central Nervous System

**DOI:** 10.1177/1759091417713905

**Published:** 2017-06-12

**Authors:** Masataka Suzuki, Nobuaki Imanishi, Masashi Mita, Kenji Hamase, Sadakazu Aiso, Jumpei Sasabe

**Affiliations:** 1 Department of Anatomy, Keio University School of Medicine, Tokyo, Japan; 2 Shiseido Co., Ltd., Tokyo, Japan; 3 Graduate School of Pharmaceutical Sciences, Kyushu University, Fukuoka, Japan

**Keywords:** D-serine, *N*-methyl-D-aspartate, glutamate receptor, Brodmann area, serine racemase, D-amino acid oxidase

## Abstract

D-serine is an endogenous ligand for *N*-methyl-D-aspartate glutamate receptors. Accumulating evidence including genetic associations of D-serine metabolism with neurological or psychiatric diseases suggest that D-serine is crucial in human neurophysiology. However, distribution and regulation of D-serine in humans are not well understood. Here, we found that D-serine is heterogeneously distributed in the human central nervous system (CNS). The cerebrum contains the highest level of D-serine among the areas in the CNS. There is heterogeneity in its distribution in the cerebrum and even within the cerebral neocortex. The neocortical heterogeneity is associated with Brodmann or functional areas but is unrelated to basic patterns of cortical layer structure or regional expressional variation of metabolic enzymes for D-serine. Such D-serine distribution may reflect functional diversity of glutamatergic neurons in the human CNS, which may serve as a basis for clinical and pharmacological studies on D-serine modulation.

## Introduction

D-serine, an enantiomer of serine, is an unusual D-amino acid *de novo* synthesized in mammals. D-serine has an enantiospecific characteristic of binding to *N*-methyl-D-aspartate (NMDA)-type glutamate receptor (NMDAR; Traynelis et al., 2010). NMDAR is one of ionotropic glutamate receptors, but distinct from the other receptors in the feature of requiring a co-agonist in addition to glutamate to be activated. D-serine binds to glycine-site of GluN1 subunit of NMDAR, which glycine also has high affinity to, whereas glutamate binds to GluN2 subunits. Although both D-serine and glycine are found at high levels in the matured rodent brain, D-serine-binding is critical to synaptic NMDAR function since genetic disruption of its synthetic enzyme, serine racemase (SR), reduces synaptic NMDAR currents and impairs synaptic plasticity in rodents (Basu et al., 2009; Benneyworth et al., 2012).

Distribution of D-serine is determined as forebrain-shifted manner by enzymatic regulation in rodents (Hashimoto et al., 1993a; Hamase et al., 1997). D-serine is converted from L-serine by a pyridoxal phosphate (PLP)-dependent enzyme SR, predominantly expressed in excitatory neurons in the central nervous system (CNS) of rodents (Miya et al., 2008; Benneyworth et al., 2012; Wolosker et al., 2016). SR catalyzes both racemization and α,β-elimination of serine enantiomers (Foltyn et al., 2005) and therefore has ability to both synthesize and degrade D-serine. Another player of D-serine regulation is a flavoenzyme, D-amino acid oxidase (DAO). DAO, found in hindbrain and spinal cord in rodents (Horiike et al., 1994; Miyoshi et al., 2011; Sasabe et al., 2014), catabolizes D-serine through oxidative deamination. D-serine is principally regulated by those two enzymes with regional difference in the brain. Knockout of SR reduces 90% of D-serine, while loss of DAO activity does not affect it in the rodent forebrain (Basu et al., 2009; Miyoshi et al., 2012), suggesting SR is a key regulator of D-serine in the area. On the other hand, in the hindbrain and spinal cord, DAO plays a critical role in determination of D-serine level because loss of DAO increases D-serine level by more than 20 times while effect of SR knockout is minimal (Hashimoto et al., 1993b; Miyoshi et al., 2012).

In contrast to its knowledge in rodents, however, regulation of D-serine in humans is less understood and potentially is not identical to that in rodents. For instance, SR is expressed not only in excitatory neurons but also in inhibitory neurons in human primary motor cortex (Balu et al., 2014) and found also in glial fibrillary acidic protein (GFAP)-positive astrocytes in the subiculum of human hippocampus (Suzuki et al., 2015). Activity-based staining of DAO shows that astrocytes in the white matter of human cerebrum has mild activity to degrade D-amino acids while such activity is absent in rodent cerebrum (Sasabe et al., 2014). D-serine level has been reported to range from 80 to 150 nmol/g (one third or half of the level in rodents) in the cerebral cortices of postmortem human brain in some studies (Hashimoto et al., 1993c; Kumashiro et al., 1995; Bendikov et al., 2007); however, it remains uncertain how regional differences affect D-serine level. As impairment of D-serine regulation has been associated with psychological and neurological disorders, such as schizophrenia (Verrall et al., 2010; Balu et al., 2013) and amyotrophic lateral sclerosis (Mitchell et al., 2010; Sasabe et al., 2012; Martinez et al., 2016), understanding of regional D-serine regulation in the human brain is crucial. In the current study, we determined detailed D-serine distribution in the human CNS using a two-dimensional high-performance liquid chromatographic (HPLC) system and compared with local expression of SR and DAO.

## Materials and Methods

### Human Samples

All procedures performed in the present study involving human participants were in accordance with the ethical standards of the institutional and national research committee and with the 1964 Helsinki declaration and its later amendments or comparable ethical standards. Informed consent was obtained prior to death from all individual participants included in the present study. Brain tissues were collected from five individuals (two males and three females) donated after death at Keio University School of Medicine. None of them died of neurological or psychological diseases. Age at death was 87.8 ± 6.1 (mean ± *SD*) years. Time at sampling after death was 26.8 ± 9.6 (mean ± *SD*) hr. Neuropathological assessment of the brains did not identify any metastasis of cancer or neurodegenerative abnormalities other than minor age-related changes. The bodies were perfused with ice-cold phosphate buffer-saline (PBS, pH 7.4) from the thoracic aorta, and then brain tissues (cerebrum was from the left hemisphere) were dissected by reference to Brodmann areas (BAs) and stored at − 80℃ until use.

### Antibodies

A rabbit polyclonal antibody to human SR, validated with Western blot of FLAG-tagged recombinant human SR protein in our previous report (Suzuki et al., 2015), was purchased from Abcam (Cambridge, UK). A goat polyclonal antibody to human DAO was from Everest biotech Ltd (Oxfordshire, UK). A rabbit polyclonal antibody to actin was obtained from Sigma Aldrich (St. Louis, MO, USA).

### Quantification of D-Serine and Glycine

D-serine or glycine concentrations in the human brain tissue were determined using a 2D-HPLC system (NANOSPACE SI-2 series, Shiseido) as previously validated and described (Miyoshi et al., 2009). Briefly, tissue homogenates were deproteinized with methanol. The amino acids in the liquid layer were derivatized with 4-fluoro-7-nitro-2,1,3-benzoxadiazole (NBD-F), separated in two tandem-connected columns (a capillary-monolithic ODS column and a narrowbore-enantioselective column, provided from Shiseido), and detected at 530 nm with excitation at 470 nm. Standard D-serine, L-serine, and glycine were obtained from Wako (Osaka, Japan).

### Western Blotting

Tissue samples were homogenized in a lysis buffer (150 mM sodium chloride, 1.0% TritonX-100, 50 mM Tris [pH 8.0], and a protease inhibitor cocktail, complete EDTA-free, Roche). The lysates were processed for SDS-PAGE. Proteins were electrically transferred to PVDF membrane, immunolabeled, and detected as previously described (Suzuki et al., 2015).

### Statistics

Prism (Graphpad software) was used for data plotting and statistical analyses. Statistical significance was determined as *p* < .05 by one-way analysis of variance (ANOVA) test (Figure 2(a) to (c)), Friedman test (Figures 3(b) and 4(b)), Kruskal–Wallis test (Figure 3(c) to (e)), or Spearman’s rank correlation coefficient (Figure 4(c)).

**Figure 1.**
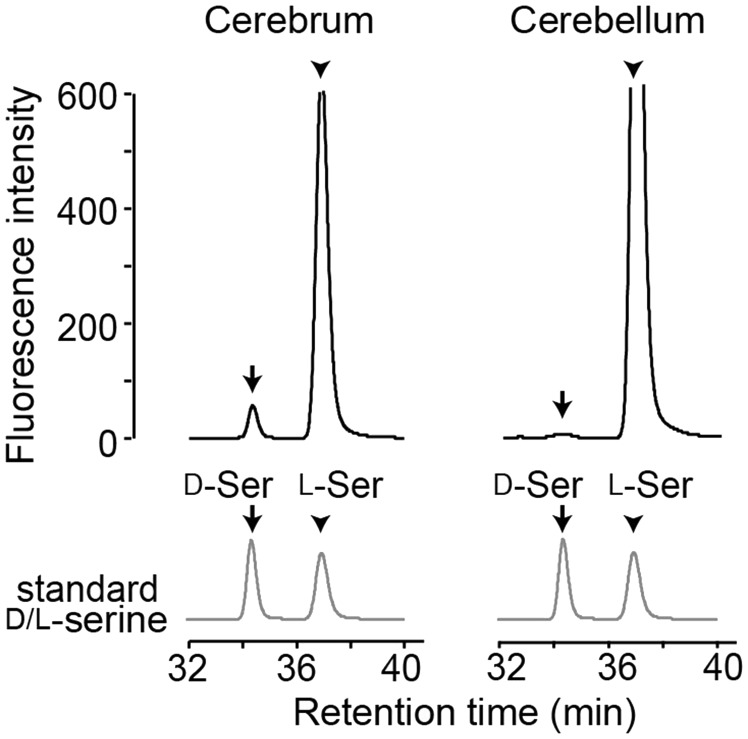
Chromatograms of serine enantiomers for the human brain tissue. Chromatograms show NBD-serine enantiomers separated with an enantioselective column in the second dimension of the 2D-HPLC (left, cerebral neocortex; right, cerebellum).

**Figure 2.**
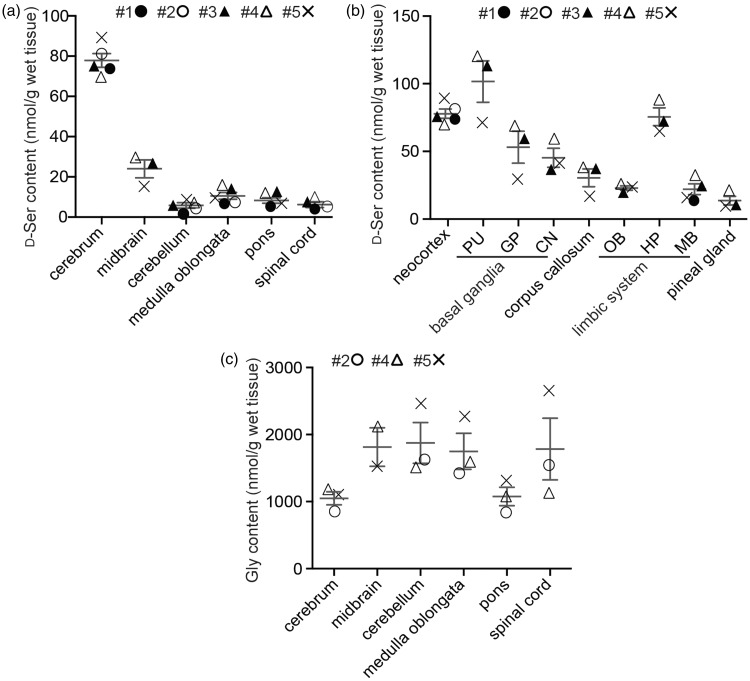
D-serine distribution in the human CNS. The concentrations of D-serine and glycine in the major parts of the brain (a, c) or D-serine in indicated parts of cerebrum (b) were quantified using 2D-HPLC (*n* = 3–5). Neocortex (BA4) was used as a representative part for the cerebrum, and substantia nigra was for the midbrain (a, c). PU, putamen; GP, globus pallidus; CN, caudate nucleus; OB, olfactory bulb; HP, hippocampus; MB, mammillary body (b). Numbers with # indicate individuals.

**Figure 3.**
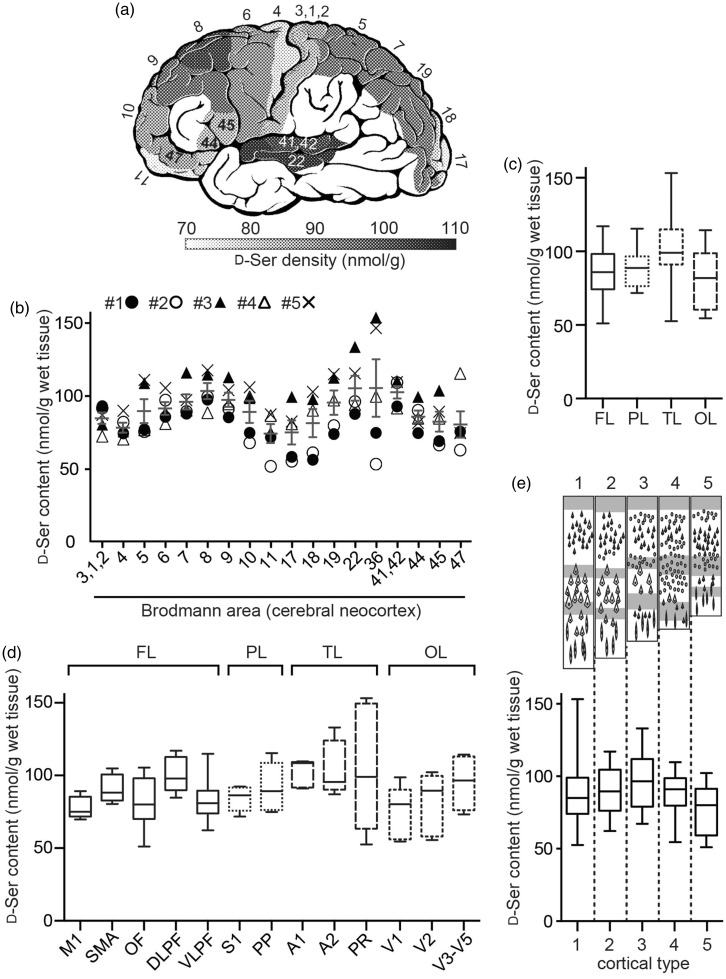
D-serine distribution within the human neocortex. (a–e) D-serine content in the gray matter of cerebral neocortex was determined using 2D-HPLC. (a) Average of D-serine content was converted into a grayscale heat map and is shown on brain cartoon numbered with Brodmann areas (*n* = 5). D-serine level in the white area without numbers was not determined. Numbers with # indicate individuals. (b) Individual levels of D-serine are plotted with average ± *SEM* for each Brodmann area. (c–e) D-serine levels in each cerebral lobe (c), functional area (d), and basic type of cortical layers (e) are shown as box plot. FL, frontal lobe; PL, parietal lobe; TL, temporal lobe; OL, occipital lobe. M1, primary motor cortex; SMA, supplementary motor area; OF, orbitofrontal cortex; DLPF, dorsolateral prefrontal cortex; VLPF, ventrolateral prefrontal cortex; S1, primary somatosentory cortex; PP, posterior parietal cortex; V1, primary visual cortex; A1, primary auditory cortex; A2, secondary auditory cortex; PR, perirhinal cortex. A scheme (upper, e) indicates typical cortical patterns. *p < .05, multiple comparisons analyzed with Kruskal–Wallis test.

**Figure 4.**
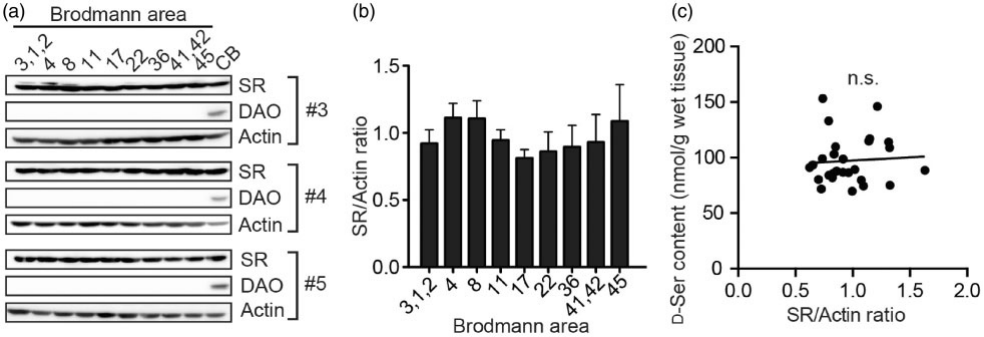
Expressions of D-serine metabolic enzymes in the neocortex. (a) Western blottings of SR, DAO, and actin using tissue lysates of indicated Brodmann’s areas and cerebellum (CB) from three individuals (#3 to #5) are shown. (b) The values represent density of the SR bands normalized to beta-actin in (a) relative to mean expression. (c) Correlation between D-serine level and relative expression of SR in each tissue area is shown. n.s., not significant, *r* = .078, analyzed by Spearman’s rank correlation coefficient.

## Results

### Determination of D-Serine Level in Brain Tissue Using 2D-HPLC

We used a two-dimensional micro-HPLC system with high sensitivity and specificity to quantify brain D-serine (Miyoshi et al., 2014). Amino acids derivatized with NBD-F in the tissue lysate were separated nonstereoselectively in the first dimension of the system. The serine fraction was further separated into D- and L-serine in the second dimension. We detected specific peaks in chromatograms of human brain tissues in accordance with those found at retention time for authentic D- and L-serine (Figure 1).

### D-Serine is Concentrated in the Human Cerebrum

First, we tested if D-serine level differs among major areas in the human CNS. Cerebrum (cerebral neocortex) contained ≈ 10-fold higher level of D-serine than brainstem, cerebellum, and spinal cord (*p* < .0001, one-way ANOVA, Figure 2(a)). The level within the cerebrum also differed among areas (*p* < .0001, one-way ANOVA, Figure 2(b)): neocortex, putamen, and hippocampus contained over 20% higher level of D-serine than cerebral average (50.2 ± 31.0 nmol/g; mean ± *SD*, total 30 parts from 3 to 5 individuals), while corpus callosum, olfactory bulb, mammillary body, and pineal gland included over 20% lower level than that. By contrast, glycine, another ligand of GluN1 subunit, was detected at levels by more than 10-fold higher than those of D-serine in the CNS, but did not show region-specific difference in the human CNS (*p* = .1855, one-way ANOVA, Figure 2(c)). These results suggest that D-serine is relatively concentrated in the areas where there are abundant glutamatergic synapses.

To further investigate D-serine distribution within the cerebral neocortex, we picked 18 BAs, which have representative neurophysiological functions. The level varied among BAs, but had a similar trend among individuals (Figure 3(a) and (b)). The D-serine level ranged from 50 to 150 nmol/g with overall average of 89.5 ± 18.9 nmol/g (mean ± *SD*, total 90 parts from 5 individuals). BA8, 22, 36, and 41/42 had over 10% higher level than the overall average, whereas BA4, 11, 17, and 47 contained over 10% lower level than that (*p* = .0013, Friedman test, Figure 3(b)).

### Heterogeneity of D-Serine Within Cerebral Neocortex is Associated With Brodmann and Functional Areas

What affects such heterogeneity in the neocortex? When we compare D-serine levels among cerebral lobes, temporal lobe contained statistically higher D-serine than frontal lobe (*p* = .045, Kruskal–Wallis test, Figure 3(c); FL is accumulation of BA4, 6, 8, 9, 10, 11, 44, 45, and 47; PL, BA3/1/2, 5, and 7; TL, BA22, 36, and 41/42; OL, BA17, 18, and 19). Since BAs have been correlated closely to diverse cortical functions, difference among functional areas was also analyzed. D-serine levels showed statistically significant variation based on cortical functions (*p* = .0232, Kruskal–Wallis test) with lowest mean value in the primary motor cortex (77.9 ± 7.6 nmol/g; mean ± *SD*) and highest in the primary auditory cortex (102.1 ± 9.5 nmol/g; mean ± *SD*; Figure 3(d); M1, BA4; SMA, BA6; OF, BA10 and 11; DLPF, BA8 and 9; VLPF, BA44, 45, and 47; S1, BA3/1/2; PP, BA5 and 7; A1, BA41/42; A2, BA22; PR, BA36; V1, BA17; V2, BA18; V3-V5, BA19).

The cerebral neocortex consists of six horizontal layers segregated principally by cell type and neuronal connections. Von Economo classified the neocortex into five fundamental cortical types by layer patterns depicted in Figure 3(e) (upper): Type 1, agranular; 2, frontal granular; 3, parietal granular; 4, polar; 5, granular (Von Economo and Koskinas, 1929). Since pyramidal neurons in neocortical layers III/V express the D-serine synthetic enzyme SR (Miya et al., 2008; Balu et al., 2014), we tested if cortical type affects D-serine level. BAs tested in this study were reclassified into five cortical types (Type 1, BA4, 6, and 36; Type 2, BA5, 7, 8, 9, 44, 46, and 47; Type 3, BA10, 19, and 22; Type 4, BA11 and 18; Type 5, BA3/1/2, 17, and 41/42). There was no statistically significant association between cortical type and D-serine level (*p* = .18, Kruskal–Wallis test; lower, Figure 3(e)), suggesting that dominance of certain cell type or neuronal connections does not simply determine D-serine level in the neocortex.

### Expressional Difference of SR Does Not Affect D-Serine Level in the Neocortex

Since SR and DAO are two major metabolic enzymes for D-serine, we further studied correlation between D-serine level and expressions of the enzymes in human CNS. SR was expressed in a comparable level in the neocortex as well as in the cerebellum, while DAO expression was dominant in the cerebellum (Figure 4(a)). Considering that D-serine level in the human cerebellum (5.9 ± 1.4 nmol/g) was ≈ 15-fold lower than that in the neocortex (Figures 2(a) and 3(b)), DAO is a dominant regulator of D-serine in human cerebellum as in rodent’s (Miyoshi et al., 2012). On the other hand, expression of SR did not differ within the neocortex (*p* = .38, Friedman test, Figure 4(b)) and, unexpectedly, difference of D-serine content among BAs also had no association with expressional variability of SR (Figure 4(c)).

## Discussion

The present study shows detailed D-serine distribution in the human CNS. D-serine is concentrated in the cerebrum, especially in the neocortex, hippocampus, and basal ganglia (putamen). Level of D-serine within the neocortex alters along with Brodmann or functional areas but is not affected by layer patterns or variation of SR expression. Since, however, we were only able to measure D-serine levels in aged brains (87.8 years in average), how developmental and aging processes affect brain D-serine levels could not be addressed in this study. Another limitation of this study is postmortem time at sampling (26.8 hr in average), and therefore, levels of amino acids might not reflect physiological conditions.

A few previous studies reported D-serine level in the human CNS (≈100 nmol/g in the frontal cortex, Hashimoto et al., 1993c; ≈100 nmol/g in the prefrontal and parietal cortices, <10 nmol/g in the cerebellum and spinal cord, Kumashiro et al., 1995; 117 ± 30 nmol/g in the parietal cortex, Bendikov et al., 2007). Our results in the present study are comparable to results in those studies and newly have shown further distribution within the cerebrum and neocortex, which confirms forebrain-shifted abundance of D-serine in humans. As D-serine has been detected at submillimolar level in the cerebrum of wide variety of mammals but not of fish, amphibians, or birds (Nagata et al., 1994; Patzold et al., 2005), forebrain-shifted abundance of D-serine in humans (Figure 2(a); Kumashiro et al., 1995) strongly supports the view that such distribution is conserved in mammals and is evolutionally distinct from the other classes of vertebrates. Although transversal use of D-serine as a neurotransmitter in vertebrates has not been fully studied, active degradation of D-serine by D-serine dehydratase (Tanaka et al., 2008), which is found in all vertebrates other than mammals, suggests that D-serine is not a principal activator of NMDAR in the nonmammalian vertebrates. One of the features of the mammalian brain structure is developed cerebral neocortex. Evolutionally obtained functional diversity of cerebral neocortex in mammals could be associated with utilization of D-serine as an activator of NMDAR, in part, because the mammalian neocortex is the largest storage of D-serine in volume in the vertebrate CNS (Figures 2 and 3(b); Nagata et al., 1994; Patzold et al., 2005) and the heterogeneity of D-serine level is linked to functional difference among areas in humans (Figure 3(d)).

We found the regional differences in D-serine level among selected 21 out of total 51 BAs of human neocortex (Figure 3(a) and (b)). The areas we studied were all from left hemisphere, were located mainly on outer surface of the frontal, parietal, and occipital lobes, and include only superior gyrus and parahippocampal gyrus in the temporal lobe. Therefore, difference between left and right hemispheres, or distribution of D-serine in internal surface of the longitudinal fissure of cerebrum or in the other parts of temporal lobe could not be determined in this study. We initially speculated that the heterogeneity of neocortical D-serine might result from the regional difference of basic layer patterns in cerebral neocortex because human SR is primarily localized to soma in pyramidal neurons with remainder in GABAergic interneurons (Balu et al., 2014). The variation of layer pattern, however, does not explain the heterogeneity of D-serine (Figure 3(e)): Type 5 cortex, which is also called as granular type with excess granule cells and minor number of pyramidal cells, includes BA3/1/2, 17, and 41/42, but BA41/42 has much higher level of D-serine than BA3/1/2 and 17 (Figure 3(b)). Given the SR localization to neuronal soma in majority of pyramidal neurons (Balu et al., 2014), our results support the view by Balu et al. (2014) that SR localization does not coincide D-serine distribution and suggest that D-serine produced in soma can be actively transported in axon along with projection and is accumulated in the destination. Although the axonal transport of D-serine or presence of D-serine in synaptic vesicles have not been identified yet, diversity of neuronal projection associated with regional functions may determine the heterogeneity of neocortical D-serine.

In the mammalian brain, SR and DAO are both crucial regulators of D-serine, although origin of about 10% of total D-serine produced in the CNS is unknown (Basu et al., 2009; Miyoshi et al., 2012). A knockout study of SR has clearly shown that mouse SR is expressed principally in soma of glutamatergic neurons and distributed in the olfactory bulb, cerebral cortex, basal ganglia (caudate-putamen), hippocampus, and substantia nigra (Miya et al., 2008). Expression of SR in astrocytes of mice had also been suggested in earlier studies (Panatier et al., 2006), but a conditional knockout study of SR in GFAP-positive astrocytes resulted in only minimal (≈10%) decrease in the hippocampus and no effects in the cortex or striatum (Benneyworth et al., 2012), suggesting that expression of SR in astrocytes is minor (cellular expression of SR is carefully discussed in other articles; Mothet et al., 2015; Wolosker et al., 2016). Contribution of astrocytes to D-serine-mediated NMDAR current is controversial since long-term potentiation at the hippocampal Schaffer collateral-CA1 synapse depends on astrocytic release of D-serine (Henneberger et al., 2010), whereas long-term potentiation at the Schaffer collateral-CA1 synapse is decreased by lack of neuronal but not astrocytic SR (Benneyworth et al., 2012). Although histological distribution of human SR is controversial due to lack of negative staining control in human studies, our results showing presence of high level of D-serine in human cerebral neocortex, basal ganglia (especially in the putamen), and hippocampus and middle level in the human olfactory bulb and midbrain (substantia nigra; Figure 2(b)) may support a view that SR distribution is macroscopically conserved between rodents and humans. Cellular distribution of SR in human CNS, however, has not been fully defined. SR is reported in glutamatergic neurons as well as in GABAergic neurons in the primary motor cortex (Balu et al., 2014) and in GFAP-positive astrocytes in the subiculum (Suzuki et al., 2015), which might be explained with variations between species, areas, or both. Such differences between rodents and humans are also known in the distribution of DAO (Sasabe et al., 2014). Activity of DAO is detected in the astrocytes of hindbrain and spinal cord in mice (Horiike et al., 1994; Schell et al., 1995; Sasabe et al., 2012), while human CNS shows mild DAO activity also in the astrocytes of white matter of cerebrum in addition to strong activity in the hindbrain and spinal cord (Sasabe et al., 2014). Since DAO has a critical role in retaining D-serine at low levels in mammals (Miyoshi et al., 2012; Sasabe et al., 2012), presence of DAO activity in human cerebral white matter may influence relatively lower cerebral D-serine level (≈100 nmol/g; Figure 3(b)) and D-/L-serine ratio (0.05–0.10, data not shown) in humans (Hashimoto et al., 1993c; Kumashiro et al., 1995; Bendikov et al., 2007) than in other mammals (D-serine, 200–500 nmol/g; D-/L-serine ratio, 0.1–0.4; Nagata et al., 1994; Patzold et al., 2005). Together with our finding that expressional variation of SR is not associated with D-serine heterogeneity in human cerebral neocortex (Figure 4), mild DAO activity in human cerebral white matter may contribute to the heterogeneity. Another factor that potentially affects brain D-serine level is expression of DAO activator (DAOA)/G72 in human CNS (Kvajo et al., 2008; Jagannath et al., 2017). DAOA, which has been identified in primates only, modulates DAO through binding to DAO, but the effect is controversial because DAO is reported to both increase (Chumakov et al., 2002; Chang et al., 2013) and decrease (Sacchi et al., 2008, 2011) the DAO activity. Therefore, with multifactorial cellular regulation of D-serine in human CNS, regional differences can be more diverse than those in rodents and comprehensive understanding awaits future studies.

Optimal NMDAR activity is crucial for neuronal homeostasis and its altered activity is implicated in multiple conditions, including aging, neurodegeneration, and neuropsychiatric disorders (Lipton, 2007; Traynelis et al., 2010). In this study, we have shown that glycine is far more abundant than D-serine throughout the human CNS (Figure 2(c)). Although the affinity of GluN1 for D-serine or glycine is very similar (Priestley et al., 1994; Madry et al., 2007), D-serine has three additional hydrogen bonds to the receptor (Furukawa and Gouaux, 2003) and may activate the NMDAR more efficiently than glycine. Intriguingly, a seminal study by Papouin et al. (2012) has demonstrated using hippocampal Schaffer collateral-CA1 synapse that D-serine would gate preferentially the synaptic GluN2A-NMDARs while glycine would target extrasynaptic receptors. As distribution pattern of SR corresponds closely to that of GluN2A/B subunits of NMDAR in rodents (Fukaya et al., 2003; Miya et al., 2008), abundance of GluN2A/B subunits in the neocortex of human CNS (Akbarian et al., 1996) is consistent with the relative abundance of D-serine in the area (Figure 2(b)) and widespread glycine-distribution may suggest additional roles of glycine such as through inhibitory glycine receptor. Given that D-serine is a dominant ligand at glycine-site of synaptic NMDAR in human CNS, modulation of D-serine level in human CNS could be associated with NMDAR activity and regarded as a therapeutic target based on growing evidence of D-serine involvement in CNS diseases, such as stroke (Mustafa et al., 2010; Abe et al., 2014), epilepsy (Harai et al., 2012; Klatte et al., 2013), Alzheimer’s disease (Hashimoto et al., 2004; Wu et al., 2004; Inoue et al., 2008), amyotrophic lateral sclerosis (Sasabe et al., 2007; Mitchell et al., 2010; Sasabe et al., 2012; Martinez et al., 2016), and schizophrenia (Balu et al., 2013; Balu and Coyle, 2015; Cho et al., 2016; Coyle et al., 2016). Although it is controversial if D-serine binding sites are saturated in the mammalian CNS, a number of studies suggest that the sites are not always saturated due to responsiveness by exogenous D-serine (Salt, 1989; Wood et al., 1989; Thiels et al., 1992; Tsai et al., 1998). Together with the fact that neocortical D-serine level is generally much lower in humans than in rodents, the heterogeneity of D-serine distribution in the human cerebral neocortex may influence physiological activity of NMDAR in the local areas.

Thus, our results on D-serine distribution improve understanding of regionally distinct regulation of D-serine in the human CNS and may serve as a basis for clinical and pharmacological studies on D-serine modulation in the human CNS.
